# Thermodynamics-Based Evaluation of Various Improved Shannon Entropies for Configurational Information of Gray-Level Images

**DOI:** 10.3390/e20010019

**Published:** 2018-01-02

**Authors:** Peichao Gao, Zhilin Li, Hong Zhang

**Affiliations:** 1 Department of Land Surveying and Geo-Informatics, The Hong Kong Polytechnic University, Hong Kong, China; 2 Faculty of Geosciences & Environmental Engineering, Southwest Jiaotong University, Chengdu 611756, China

**Keywords:** Shannon entropy, information entropy, information content, configurational information

## Abstract

The quality of an image affects its utility and image quality assessment has been a hot research topic for many years. One widely used measure for image quality assessment is Shannon entropy, which has a well-established information-theoretic basis. The value of this entropy can be interpreted as the amount of information. However, Shannon entropy is badly adapted to information measurement in images, because it captures only the compositional information of an image and ignores the configurational aspect. To fix this problem, improved Shannon entropies have been actively proposed in the last few decades, but a thorough evaluation of their performance is still lacking. This study presents such an evaluation, involving twenty-three improved Shannon entropies based on various tools such as gray-level co-occurrence matrices and local binary patterns. For the evaluation, we proposed: (a) a strategy to generate testing (gray-level) images by simulating the mixing of ideal gases in thermodynamics; (b) three criteria consisting of validity, reliability, and ability to capture configurational disorder; and (c) three measures to assess the fulfillment of each criterion. The evaluation results show only the improved entropies based on local binary patterns are invalid for use in quantifying the configurational information of images, and the best variant of Shannon entropy in terms of reliability and ability is the one based on the average distance between same/different-value pixels. These conclusions are theoretically important in setting a direction for the future research on improving entropy and are practically useful in selecting an effective entropy for various image processing applications.

## 1. Introduction

Image quality assessment plays a fundamental role in the field of digital image processing [1,2,3,4,5,6], where it is useful in monitoring the quality of image systems, benchmarking image processing applications, and optimizing image processing algorithms [7,8]. The most reliable approach to assess image quality is a visual observation with the naked eye [9], but this approach depends largely on individual interpretations of quality and is thus subjective. For objective image quality assessment, one simple and widely used approach is to quantify the amount of (syntactic) information contained in an image using information-theoretic measures [10,11,12,13,14,15,16,17]. It is believed that the more information an image contains, the better the quality of the image is [12].

The most basic information-theoretic measure is entropy, which was proposed by Shannon [18] in the area of telecommunication. Shannon entropy (also called information entropy) is widely recognized as a cornerstone of information theory [19], and it has been used in various fields such as physics e.g., [20], chemistry e.g., [21], and biology e.g., [22]. Although Shannon entropy was originally used to quantify the information (i.e., disorder) of a one-dimensional message (e.g., a telegram message consisting of a series of letters), it has also been actively utilized as a measure of information content for gray-level (or grayscale) images, which can be considered as two-dimensional messages, in various applications including registration, segmentation, and fusion [23,24,25,26,27,28].

**Figure 1:**
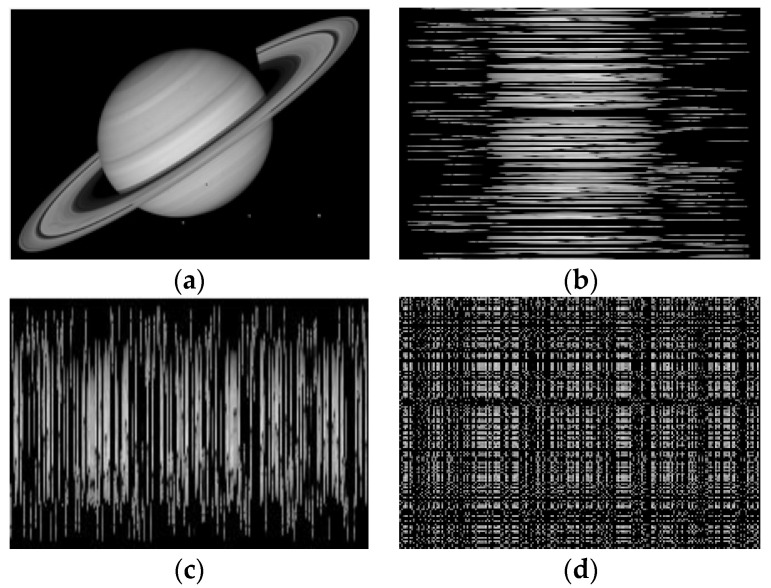
Four images with the same composition, but different configurations of pixels. These four images contain a Saturn image (**a**); two images generated by randomizing either the rows (**b**); or the columns (**c**) of the Saturn image; and an image generated by randomizing both the rows and the columns of the Saturn image (**d**). The Shannon entropies of all these four images (**a**–**d**) are 3.96 bits.

However, the information contained in a gray-level image (hereafter simply image) cannot be fully characterized by Shannon entropy as it only captures the image’s compositional (or non-spatial) information such as the proportions and gray values of different pixels. The configurational (or spatial) information (i.e., the spatial distribution of pixels) of an image is ignored by Shannon entropy; see an example in Figure 1, where four images with different configurations of pixels have the same Shannon entropy. In fact, this problem of Shannon entropy has been pointed out by a number of researchers [29,30,31,32,33,34], questioning the applicability of Shannon entropy as a measure of information content of two-dimensional messages such as images, maps, and digital elevation models.

To overcome the above problem, many improved Shannon entropies have been proposed in the last few decades to quantify the configurational information of an image, or, more specifically, the configurational disorder (or configuration) of pixels in an image. Nevertheless, to the best of our knowledge, no comparative study has been conducted concerning the performance of different improved Shannon entropies. More seriously, in the original papers on improved Shannon entropies, evaluations were either omitted e.g., [35] or simply performed in one of the two following ways:

- to check whether the improved Shannon entropies of a few examples of spatial patterns are different e.g., [36], or
- to examine whether the performance of a Shannon entropy-based image processing algorithm is improved e.g., [37].

Such evaluations are incomprehensive and sometimes case dependent. This study aims to systematically evaluate and compare the performance of improved Shannon entropies.

The remainder of this article is organized as follows: Section 2 presents a critical review of Shannon entropy and its improvements. Section 3 describes the design of the experiments to evaluate the performance of various improved Shannon entropies. A strategy to simulate configurational disorder (used as the experimental data) and a set of measures for evaluation is also proposed in this section. Then, Section 4 reports the experimental results and the analysis in terms of validity, reliability, and ability. It is found that the improved Shannon entropies based on local binary patterns are invalid for use in quantifying the configurational information of images, and the best variant of Shannon entropy in terms of reliability and ability is the one based on the average distance between same/different-value pixels. Section 5 presents a further discussion, followed by some concluding remarks in Section 6.

## 2. A Critical Review of Improved Entropies

The formula of Shannon entropy (referred to as *Sh48*, which is a short name formed from the letters of the author’s surname and digits of the year of publication) is given as follows:

(1) $$H\left(X\right)=-\overset{n}{\underset{i=1}{\sum }}P\left(x_{i}\right)\log_{2}P\left(x_{i}\right)$$

where X is a discrete random variable with possible values of $\left\{x_{1},x_{2},\cdots ,x_{i},\cdots ,x_{n}\right\}$, and $P\left(x_{i}\right)$ is the probability of X taking the value of $x_{i}$. When *Sh48* is used for an image, X denotes the pixel of the image, and $P\left(x_{i}\right)$ is the proportion of the pixels with a gray value of $x_{i}$.

To make Shannon entropy capable of quantifying the configurational information of an image, one should first characterize the configuration of image pixels using a certain tool and then reflect the characterization in the computation of Shannon entropy. Six tools have been used in the literature, leading to six categories of improved Shannon entropies as follows:

1. Entropies based on the gray-level co-occurrence matrix of an image;
2. Entropies based on the gray-level variance of the neighborhood of a pixel;
3. Entropy based on the Sobel gradient of a pixel;
4. Entropy based on the local binary pattern of an image;
5. Entropy based on the Laplacian pyramid of an image; and
6. Entropy based on the distance between pixels of the same/different value.

These six categories are reviewed in the remainder of this section.

### 2.1. Entropies Based on the Gray-Level Co-Occurrence Matrix of an Image

**Figure 2:**
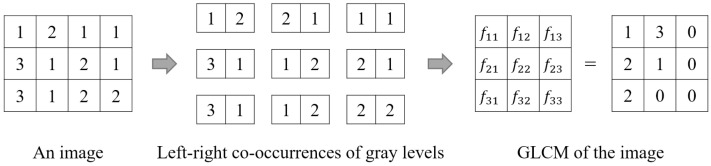
Process of obtaining the gray-level co-occurrence matrix (GLCM) of an image.

The gray-level co-occurrence matrix (GLCM) was first proposed by Haralick, et al. [35] and is still widely used in image processing e.g., [38,39]. The basic idea behind it is the co-occurrence of two gray levels in an image. For example, there are nine co-occurrences of gray levels when scanning the image in Figure 2 from left to right and pixel by pixel. The GLCM of the image, also shown in Figure 2, is a matrix that records the frequency of such co-occurrence of every two gray levels. In this example, the element $f_{ij}$ of the matrix indicates that the j-th gray level occurs $f_{ij}$ time (s) at the immediate right of the i-th gray level.

**Figure 3:**
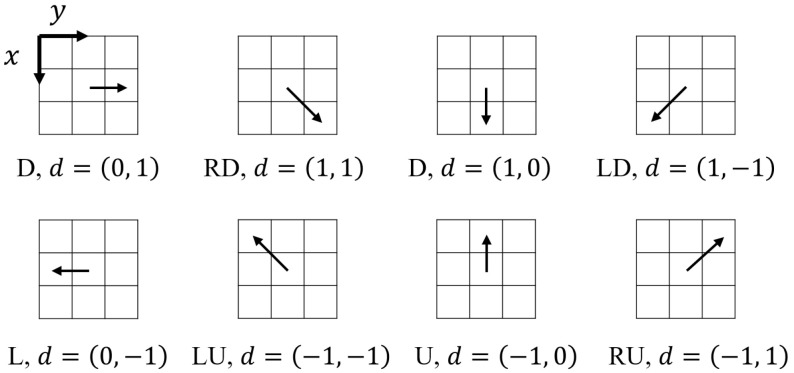
Displacement operators that can be used to generate a GLCM along eight directions, i.e., right (R), right-down (RD), down (D), left-down (LD), left (L), left-up (LU), up (U), and right-up (RU).

Formally, the GLCM of a M×N image with L gray levels is given as a L×L matrix, $\left\{f_{ij}|1\leq i\leq L,1\leq j\leq L\right\}$, the element of which is computed according to Equation (2):

(2) $$f_{ij}=\overset{M}{\underset{m=1}{\sum }}\overset{N}{\underset{n=1}{\sum }}\{\begin{array}{cc} 1 & I\left(m,n\right)=G\left(i\right)\;\mathrm{and}\;I\left(m+∆x,n+∆y\right)=G\left(j\right)\\ 0 & \mathrm{otherwise} \end{array}$$

where G(x) is the value of the x-th gray level in the image, I(m,n) denotes the gray value of the pixel located at (m,n), and (∆x,∆y) is a pair of pre-set parameters called the displacement operator (denoted as d). Haralick, et al. [35] provided a total of eight displacement operators (Figure 3), which can be used to generate GLCMs along eight different directions, i.e., right (R), right-down (RD), down (D), left-down (LD), left (L), left-up (LU), up (U), and right-up (RU).

Based on the GLCM of an image, Haralick, et al. [35] developed a new method to compute Shannon entropy (denoted as *Ha73*), as shown in Equation (3). Note that according to this equation, a total of eight GLCM-based improved Shannon entropies can be obtained because there are eight directions (R, RD, D, LD, L, LU, U, and RU; see Figure 3) along which a GLCM can be generated. In this study, these eight improved Shannon entropies are referred to as *Ha73-R*, *Ha73-RD*, *Ha73-D*, *Ha73-LD*, *Ha73-L*, *Ha73-LU*, *Ha73-U*, and *Ha73-RU*, respectively:

(3) $$Ha73=-\underset{i}{\sum }\underset{j}{\sum }\left(\frac{f_{ij}}{\sum_{i}\sum_{j}f_{ij}}\right)\cdot \log\left(\frac{f_{ij}}{\sum_{i}\sum_{j}f_{ij}}\right)$$

It should be pointed out that all eight improved Shannon entropies by Haralick, et al. [35] are computed based on the GLCM generated along only one direction. One may argue that the configurational information quantified by such Shannon entropies is incomplete. For this reason, three other methods to generate a GLCM were proposed for the computation of a GLCM-based improved Shannon entropy using Figure 3.

(1) GLCM generated along two directions

In computing a GLCM-based improved Shannon entropy, Pal and Pal [40] proposed generating a GLCM with displacement operators along two directions, namely “R” and “D”. In other words, the element ($f_{ij}$) of such a GLCM is derived using Equations (4)–(6). The resultant improved Shannon entropy is referred to as *PP89* in this study:

(4) $$f_{ij}=\overset{M}{\underset{m=1}{\sum }}\overset{N}{\underset{n=1}{\sum }}\left(\delta_{1}\left(m,n\right)+\delta_{2}\left(m,n\right)\right)$$

(5) $$\delta_{1}\left(m,n\right)=\{\begin{array}{cc} 1 & I\left(m,n\right)=G\left(i\right)\;\mathrm{and}\;I\left(m+1,n\right)=G\left(j\right)\\ 0 & \mathrm{otherwise} \end{array}$$

(6) $$\delta_{2}\left(m,n\right)=\{\begin{array}{cc} 1 & I\left(m,n\right)=G\left(i\right)\;\mathrm{and}\;I\left(m,n+1\right)=G\left(j\right)\\ 0 & \mathrm{otherwise} \end{array}$$

(2) GLCM generated along eight directions

Abutaleb [41] proposed considering all eight directions when generating a GLCM with an image. In his method, the element ($f_{ij}$) of the GLCM of an image is computed using Equations (7) and (8). Note that in this way, the term “gray-level co-occurrence” in “GLCM” is actually redefined to be the co-occurrence of the gray level of a pixel and the average gray level of the pixel’s eight neighbors. The resultant improved Shannon entropy is referred to as *Ab89*:

(7) $$f_{ij}=\overset{M}{\underset{m=1}{\sum }}\overset{N}{\underset{n=1}{\sum }}\{\begin{array}{cc} 1 & I\left(m,n\right)=G\left(i\right)\;\mathrm{and}\;Ave\left(m,n\right)=G\left(j\right)\\ 0 & \mathrm{otherwise} \end{array}$$

(8) $$Ave\left(m,n\right)=\frac{1}{8}\left(\overset{1}{\underset{k=-1}{\sum }}\overset{1}{\underset{l=-1}{\sum }}I\left(k,l\right)-I\left(m,n\right)\right)$$

(3) GLCM generated along four directions

Brink [42] proposed the use of only four directions containing “R”, “RD”, “D”, and “LD” when computing the GLCM-based Shannon entropy (referred to as *Br95*) of an image; that is, each element of the GLCM of an image is derived using Equations (9)–(13). In this way, the GLCM employed by Brink [42] is based on the asymmetrical neighborhood of a pixel, rather than the symmetrical neighborhood used by Abutaleb [41]. It is worth noting that such asymmetrical neighborhoods are now widely used in generating the GLCM of an image [43]:

(9) $$f_{ij}=\overset{M}{\underset{m=1}{\sum }}\overset{N}{\underset{n=1}{\sum }}\overset{4}{\underset{p=1}{\sum }}\delta_{p}\left(m,n\right)$$

(10) $$\delta_{1}\left(m,n\right)=\{\begin{array}{cc} 1 & I\left(m,n\right)=G\left(i\right)\;\mathrm{and}\;I\left(m,n+1\right)=G\left(j\right)\\ 0 & \mathrm{otherwise} \end{array}$$

(11) $$\delta_{2}\left(m,n\right)=\{\begin{array}{cc} 1 & I\left(m,n\right)=G\left(i\right)\;\mathrm{and}\;I\left(m+1,n+1\right)=G\left(j\right)\\ 0 & \mathrm{otherwise} \end{array}$$

(12) $$\delta_{3}\left(m,n\right)=\{\begin{array}{cc} 1 & I\left(m,n\right)=G\left(i\right)\;\mathrm{and}\;I\left(m+1,n\right)=G\left(j\right)\\ 0 & \mathrm{otherwise} \end{array}$$

(13) $$\delta_{4}\left(m,n\right)=\{\begin{array}{cc} 1 & I\left(m,n\right)=G\left(i\right)\;\mathrm{and}\;I\left(m+1,n-1\right)=G\left(j\right)\\ 0 & \mathrm{otherwise} \end{array}$$

### 2.2. Entropies Based on the Gray-Level Variance of Neighborhoods of a Pixel

**Figure 4:**
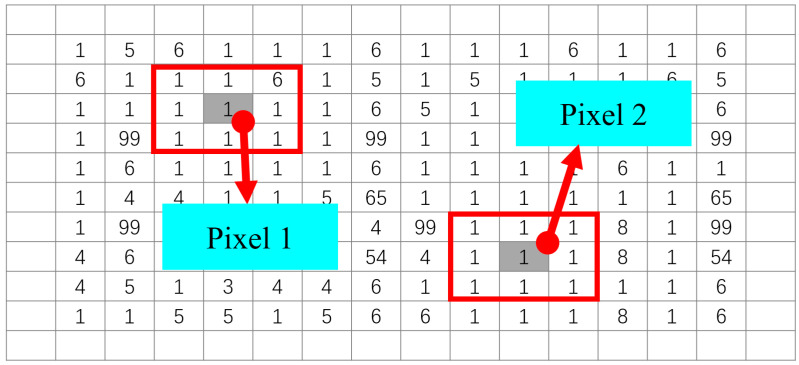
Two pixels with the same gray level but different neighborhoods.

The configuration of pixels of an image can also be captured by the gray-level variance (GLV) computed for the neighborhood of each pixel. This is because two pixels with the same gray value, but different neighbors are likely to have different GLVs, as shown in Figure 4. In the literature, there are two improved Shannon entropies based on the GLVs of pixels.

The first GLV-based improved Shannon entropy (referred to as *Br96*) was proposed by Brink [44] in the form of Equations (14)–(16):

(14) $$Br96=-\overset{n}{\underset{i=1}{\sum }}p_{i}\cdot \log\left(\frac{p_{i}}{m_{i}}\right)$$

(15) $$m_{i}=1+\delta_{i}=1+\underset{i\in N_{3}}{\sum }\frac{{\left(g_{i}-\mu_{N_{3}}\right)}^{2}}{9}$$

(16) $$p_{i}=\frac{g_{i}}{G}=g_{i}/\overset{n}{\underset{i=1}{\sum }}g_{i}$$

where n is the number of pixels in an image; $N_{3}$ is the 3×3 neighborhood (including the pixel itself) of a pixel; $\mu_{N_{3}}$ is the average gray value of pixels in $N_{3}$; $\delta_{i}$ is the GLV of $N_{3}$; and $g_{i}$ is the gray value of pixel 𝑖. Note that in this improved Shannon entropy, the probability $p_{i}$ is computed for each pixel rather than for each gray level in the original Shannon entropy.

The other GLV-based improved Shannon entropy (referred to as *Qu12-V*) was proposed by Quweider [37] and computed using the following equations:

(17) $$Qu12-V=-\overset{n}{\underset{l=1}{\sum }}p_{l}\cdot \log\left(\frac{p_{l}}{m_{l}}\right)$$

(18) $$Qu12-V=-\overset{n}{\underset{l=1}{\sum }}p_{l}\cdot \log\left(\frac{p_{l}}{m_{l}}\right)$$

where n is the number of gray levels in an image; l denotes a gray level; $\Omega_{l}$ is the collection of coordinates of pixels with a gray value of l; $\left|\Omega_{l}\right|$ is the number of elements in $\Omega_{l}$; and δ(i,j) is the GLV of the 3×3 neighborhood of pixel (i,j). Note that the probability $p_{l}$ in Equation (17) is computed for all pixels at the same gray level, rather than for a single pixel in Equation (14). In the literature, the parameter $m_{l}$ is commonly referred to as the busyness or activity of the gray level l [37,45].

### 2.3. Entropy Based on the Sobel Gradient of a Pixel

**Figure 5:**
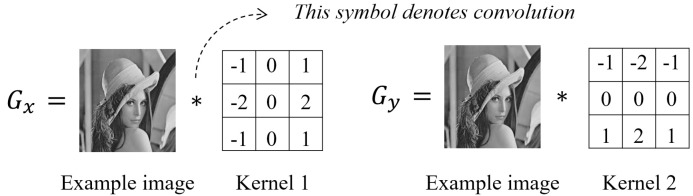
Two kernels defined by the Sobel operator. These kernels are used to convolve an image and the results of convolution (i.e., $G_{x}$ and $G_{y}$) are useful in computing Sobel gradients.

Different configurations of pixels may lead to different edges, which can be detected by computing the gradient of each pixel [46,47]. One of the commonly used tools to determine the gradient of a pixel is the Sobel operator [48], which consists of two 3×3 kernels (Figure 5) used to convolve an image (denote the convolved images as $G_{x}$ and $G_{y}$, respectively).

The first kernel aims to detect the edges of the image in the horizontal direction, whereas the second kernel operates in the vertical direction. Based on $G_{x}$ and $G_{y}$, the (Sobel) gradient of a pixel (i,j) is computed as follows:

(19) $$G\left(i,j\right)=\sqrt{{\left(G_{x}\left(i,j\right)\right)}^{2}+{\left(G_{y}\left(i,j\right)\right)}^{2}}$$

Quweider [37] proposed a Sobel gradient-based Shannon entropy, referred to as *Qu12-G*. This entropy is also computed using Equation (17), but the busyness $m_{l}$ in Equation (17) is redefined as the average Sobel gradient of all pixels with a gray value of l, as shown in Equation (20):

(20) $$m_{l}=\frac{1}{\left|\Omega_{l}\right|}\cdot \underset{\left(i,j\right)\in \Omega_{l}}{\sum }G\left(i,j\right)$$

where $\Omega_{l}$ denotes the collection of coordinates of pixels with a gray value of l; $\left|\Omega_{l}\right|$ is the number of elements in $\Omega_{l}$; and G(i,j) is the Sobel gradient computed according to Equation (19).

### 2.4. Entropy Based on the Local Binary Pattern of an Image

**Figure 6:**
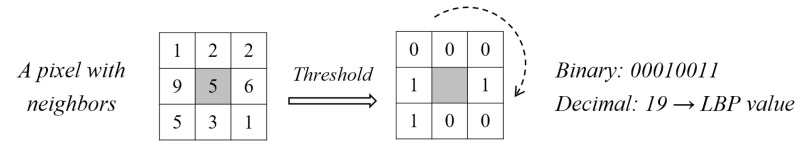
A pixel and its local binary pattern (*LBP*) value.

A specific configuration of pixels may form a specific local binary pattern (LBP), which is a popular local texture descriptor that was first introduced by Ojala et al. [49] and is widely used in image analysis e.g., [50,51]. The LBP of an image is expressed as a series of integers called the LBP values, which are assigned to each pixel of an image. The procedure to determine the LBP value of a pixel is as follows (an example is shown in Figure 6).

1. Read the gray value (y) of the pixel and that of the pixel’s eight immediate neighbors from the left top in clockwise order (denoted as $x_{0},x_{1},\cdots ,x_{7}$).
2. Create an 8-digit binary number, $b_{0}b_{1}b_{2}b_{3}b_{4}b_{5}b_{6}b_{7}$, where $b_{i}$ (0≤i≤7) is a binary digit with a value of either 0 or 1.
3. Compare each neighbor to the pixel; set $b_{i}=1$ if $x_{i}>y$. Otherwise, set $b_{i}=0$.
4. Convert the binary number to its decimal equivalent, which is the *LBP* value of the pixel.

An *LBP*-based Shannon entropy (referred to as *Qu12-L*) was suggested by Quweider [37] in the same form as Equation (17), but the busyness $m_{l}$ in Equation (17) is computed as follows:

(21) $$m_{l}=\frac{1}{\left|\Omega_{l}\right|}\cdot \underset{\left(i,j\right)\in \Omega_{l}}{\sum }LBP\left(i,j\right)$$

where LBP(i,j) is the *LBP* value of pixel (i,j), and $\Omega_{l}=\left\{\left(i,j\right)|I\left(i,j\right)=l\right\}$ is the collection of coordinates of pixels with a gray value of l.

### 2.5. Entropy Based on the Laplacian Pyramid of an Image

**Figure 7:**
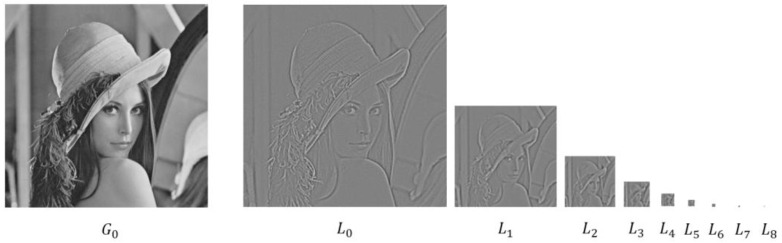
The gray-level Lena image $G_{0}$ (256×256 pixels) and its Laplacian pyramid, which consists of nine levels: $L_{0},L_{1},L_{2},L_{3},L_{4},L_{5},L_{6},L_{7}$, and $L_{8}$.

Rakshit and Mishra [52] pointed out that the configuration of pixels in an image can be captured by its Laplacian pyramid, which is proposed by Burt and Adelson [53] and has been widely used for image analysis [54]. The Laplacian pyramid is a type of multi-scale representation for images, and it is constructed by decomposing an image into multiple scales (or levels, denoted as $L_{0},L_{1},\cdots ,L_{i},\cdots ,L_{n-1},L_{n}$), as shown in Figure 7. 

In a Laplacian pyramid, the size of the first level ($L_{0}$) is the same as that of the original image, whereas the size of each of the other levels is half of that of its previous level (please see [55] for more technical details on the Laplacian pyramid).

The assumption behind Rakshit and Mishra [52]’s argument is that two different images with the same composition of pixels are likely to have different Laplacian pyramids; thus, the difference in the configuration of pixels in the two images can be reflected in measures based on the Laplacian pyramid. Based on this assumption, they proposed an improved Shannon entropy (referred to as *RM06*) that is computed as follows:

(22) $$RM06=\overset{n}{\underset{i=0}{\sum }}H\left(L_{i}\right)$$

where $H\left(L_{i}\right)$ is the Shannon entropy of the i-th level (denoted as $L_{i}$ where i=0,1,⋯,n) of the Laplacian pyramid of an image.

### 2.6. Entropy Based on the Average Distance between Same/Different-Value Pixels

The configuration of pixels (or geographic features in general) determines their correlation, which can be estimated, according to Claramunt [36], by using the Euclidean distance. Following this line of thought, Claramunt [36] proposed an improved Shannon entropy based on the distance between two pixels, or the geographic features in general.

The distance between two pixels, as pointed out by Claramunt [36], can be considered as the key factor in determining the correlation between them, because the *First Law of Geography* [56] states that “everything is related to everything else, but near things are more related than distant things” [57]. This key, according to Claramunt [36], should also be used in determining the correlation among all the pixels of an image, or the configurational disorder of an image. He assumed that the degree of the configurational disorder of an image would decrease if the average distance between every two pixels of the same gray value (or same-value pixels in short) becomes shorter and/or the average distance between every two pixels of different gray values (or different-value pixels) becomes longer. With this assumption, Claramunt [36] proposed an improved Shannon entropy (referred to as *Cl05*) which is computed by the following three equations:

(23) $$Cl05=-\overset{n}{\underset{i=1}{\sum }}\frac{d_{s}\left(i\right)}{d_{d}\left(i\right)}\cdot p_{i}\cdot \log p_{i}$$

(24) $$d_{s}\left(i\right)=\{\begin{array}{cc} \frac{1}{N_{i}\cdot \left(N_{i}-1\right)}\overset{N_{i}}{\underset{j=1,j\in C_{i}}{\sum }}\overset{N_{i}}{\underset{k=1,k\neq j}{\sum }}d_{jk} & N_{i}>1\\ \lambda & N_{i}=1 \end{array}$$

(25) $$d_{d}\left(i\right)=\{\begin{array}{cc} \frac{1}{N_{i}\cdot \left(N-N_{i}\right)}\overset{N_{i}}{\underset{j=1,j\in C_{i}}{\sum }}\overset{N-N_{i}}{\underset{k=1,k\notin C_{i}}{\sum }}d_{jk} & N_{i}\neq N\\ \lambda & N_{i}=N \end{array}$$

where i denotes the i-th gray level; and n and N are the total number of gray levels and that of pixels, respectively. $p_{i}$, $N_{i}$, and $C_{i}$ are the proportion, the total number, and the collection of pixels at the i-th gray level, respectively. j and k denote the j-th and k-th pixel in $C_{i}$, respectively, and the Euclidean distance between them is denoted by $d_{jk}$. λ is a pre-set parameter taking a small value such as 0.1 or 0.2.

The nature of the $d_{s}\left(i\right)$ computed using Equation (24) is the average of the distances between every two pixels at the i-th gray level. Therefore, $d_{s}$ is termed the average distance between the same-value pixels in this study. In contrast, $d_{d}\left(i\right)$ is actually the average of the distances between each of the pixels at the i-th gray level and each of the pixels at the other gray levels, so $d_{d}$ is referred to as the average distance between the different-value pixels. In the work by Leibovici, et al. [58], $d_{s}/d_{d}$ is termed *discriminant ratio*.

It is worth noting that, although a comprehensive evaluation is lacking, *Cl05* has found some applications in geographic information science. Examples of these applications include spatial data classification [59] and clustering [60].

## 3. Design of the Thermodynamics-Based Evaluation

The basic idea of the evaluation is to compute the values of an improved Shannon entropy for a sequence of increasingly configuration-disordered images and then to examine whether these values capture the increasing disorder or not. However, there is no standard sequence of images that are increasingly disordered in terms of configuration. In this section, a thermodynamics-based strategy is first proposed and used to generate such images. Then, the criteria for the evaluation are defined and measures for each criterion are developed.

### 3.1. A Thermodynamics-Based Strategy for Generating Testing Images

To obtain a sequence of increasingly configuration-disordered images, one natural strategy is to generate a group of images with the same composition of pixels and then rank these images according to their degrees of configurational disorder. Such a strategy requires a measure of (configurational) disorder that can be employed to rank different configuration-disordered images, or configurational disorders in general. However, the long-used standard measure of disorder is Shannon entropy itself [61,62], but, as mentioned in the introduction, its value is not related to configurational disorders.

**Figure 8:**
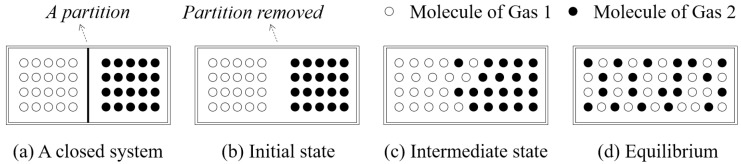
The mixing of two ideal gases in a closed system. (**a**) Two ideal gases are separated by a partition in a container; (**b**) The partition is removed and the two ideal gases begin to mix together; (**c**) An intermediate state of the mixing; (**d**) The final state, equilibrium, of the mixing.

To escape the above paradox, the origin of the entropy concept, thermodynamics, was revisited in this study. In thermodynamics, the terms entropy and disorder are used interchangeably [63]. The classical example of increasing disorder is the mixing of ideal gases [64], as shown in Figure 8. In this example, two ideal gases are initially separated by a partition in a closed system (Figure 8a), and then they mix together because the partition is removed (Figure 8b–d). During the mixing process, the disorder/entropy of the system increases logarithmically until the system achieves equilibrium [65], at which time the disorder/entropy reaches its maximum value.

One possible strategy for generating a sequence of increasingly configuration-disordered images is to simulate this classical example in thermodynamics, i.e., the mixing of ideal gases. To this end, a simulation strategy, referred to as the thermodynamics-based strategy, was proposed in this study. The strategy works with a user-supplied image, referred to as a “seed” (image), which is regarded as the initial state of a closed system. In the strategy, pixels of the seed image are regarded as gas molecules, whose “mixing” is simulated using the following iterative algorithm:

1. Get the size, r×c, of the seed image, which is taken as the output of Iteration 0.
2. Randomly select (r×c)/2 pixels in the resultant image of the previous iteration.
3. Exchange the position of each of the selected pixels and a randomly selected neighboring pixel.
4. Output the resultant image as the result of the current iteration of mixing.
5. Go back to Step 2 until the number of iterations reaches some threshold.

### 3.2. A Set of Testing Images Generated Using the Proposed Strategy

**Figure 9:**
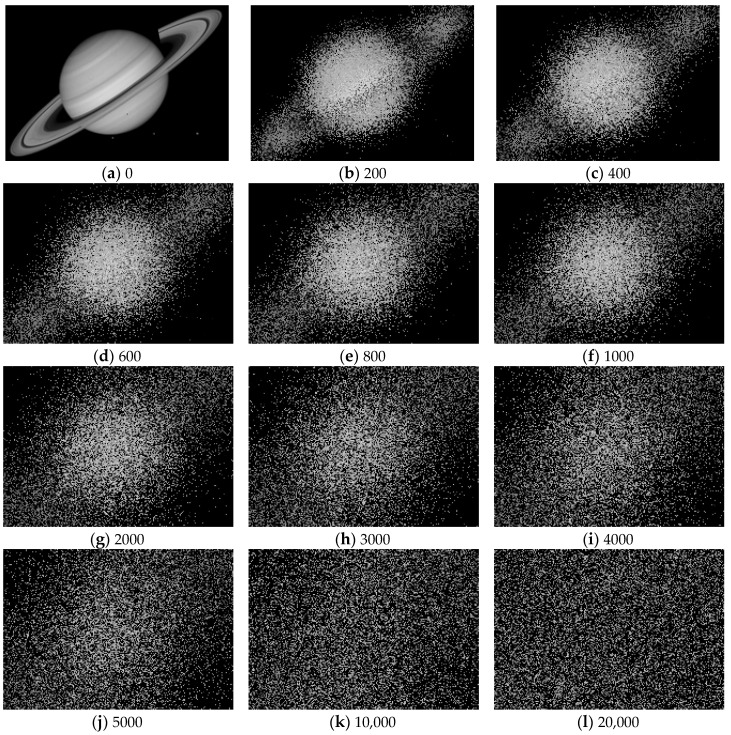
A natural image (**a**) and eleven configuration-disordered images (**b**–**l**) generated based on it using the proposed thermodynamics-based strategy. Note that a total of 20,000 configuration-disordered images were generated, but only a few of them are displayed here. The displayed images are outputs of Iterations 200 (**b**); 400 (**c**); ...; 1000 (**f**); 2000 (**g**); ..., 5000 (**j**); 10,000 (**k**); and 20,000 (**l**).

Using the thermodynamics-based strategy, a set of testing images were generated in this study. The testing image set is a sequence of increasingly configuration-disordered images generated using a natural image (Figure 9a) as the seed. This seed image contains 150×215 pixels, with values ranging from 0 to 215. The threshold in implementing the thermodynamics-based strategy was determined using the following procedure:

1. Set its initial value to a large enough number (e.g., 100,000) to obtain numerous outputs.
2. View the outputs of the 10,000 ×k-th (k=1,2,3,⋯) iterations with the naked eye, and select one from these viewed outputs as the “total disorder”.
3. Set the final value of the threshold to the number of iterations of the “total disorder”.

Following the preceding procedure, the threshold was determined as 20,000. In other words, the testing image set contains 20,000 increasingly configuration-disordered images (see a few of these images in Figure 9b–l), each of which is the output of the i-th (i=1,2,3,⋯,20,000) iteration of mixing using the natural image (Figure 9a) as the seed. 

Some readers may wonder what the mixing result is like after 20,000 iterations. Our experiment, consisting of 100,000 iterations of mixing, showed that there was little visual difference between two resultant images after 20,000 iterations (the results of 100,000 iterations are available from the authors upon request).

### 3.3. Criteria and Measures for Evaluation

Three criteria are defined in this section for evaluating the improved Shannon entropies, i.e., their validity, reliability, and ability to capture configurational disorder. In addition to the definition of these criteria, three measures were developed to assess the fulfillment of each criterion.

(1) Validity and its measure

Validity is the most important criteria; it indicates “whether the instrument is actually measuring the concept it claims to measure” [66]. In this study, the validity of an improved Shannon entropy refers to whether the entropy really captures configurational disorder or not. In dealing with the testing images, the values of a valid improved Shannon entropy for these images should exhibit a logarithmic trend over the iterations of mixing. Such a trend is a characterization of the logarithmic growth of the degree of the configurational disorder of pixels—as simulations of gas molecules in mixing—in the iterations. The measure of validity, referred to as V, is qualitatively defined as follows:

(26) $$V=\{\begin{array}{cc} Yes & r^{2}\geq thre\\ No & r^{2}<thre \end{array}$$

where yes means valid, and no indicates invalid. The parameter thre is a pre-set threshold, and $r^{2}$ is the coefficient of determination obtained when performing a least-squares regression between (a) the values of an improved Shannon entropy for the testing images and (b) the iterations of mixing, using a logarithmic model. The value of $r^{2}$ indicates the goodness of fit of a regression model to data [67], so in the context of this study it demonstrates whether the logarithmic trend shown by these values over the iterations of mixing is strong. In this study, the value of thre was set as 50% because a regression model can usually be regarded as a good fit, if $r^{2}$ is greater than a half [68].

(2) Reliability and its measure

**Figure 10:**
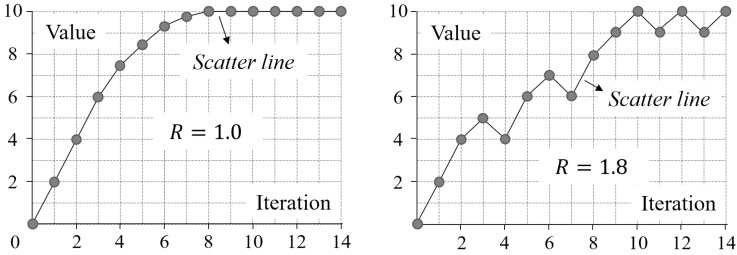
Two example scatter lines and their corresponding values of the reliability measure (R).

The reliability of a measure refers to “whether something is being measured consistently” [69]. The meaning of reliability is two-fold. First, a reliable measure “produces the same results when used repeatedly to measure the same thing” [70]. Second, the values of a reliable measure for two similar things are close. In the second sense, if an improved Shannon entropy is reliable, the difference between its values for the configuration-disordered images at two consecutive iterations of mixing should be tiny. In other words, if the values of a reliable improved Shannon entropy for the testing images are shown in a scatter plot, the polyline (hereafter referred to as the scatter line) connecting every two consecutive scatter points should be smooth (see [71,72] for more information on scatter plots). The measure of reliability, referred to as R, is quantitatively defined as follows:

(27) $$R=\left(\overset{n-1}{\underset{i=1}{\sum }}\left(v_{i+1}-v_{i}\right)\right)/\left(max-min\right)$$

where $v_{i}$ is value of an improved Shannon entropy for the configuration-disordered image at the i-th iteration of mixing (i=1,2,3,⋯,n); n is the total number of iterations; and max and min are the maximum and minimum of all ($v_{i}$)s, respectively. It can be seen from Equation (27) that R is the ratio of (a) the cumulative growth in value of an improved Shannon entropy for the configuration-disordered images from the first iteration to the last to (b) the value range of this entropy for the images of all iterations. The smaller this ratio, the smoother the scatter line (see an example in Figure 10), and the more reliable the improved Shannon entropy.

(3) Ability and its measure

**Figure 11:**
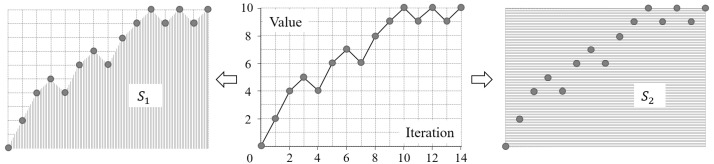
An example scatter plot and the two corresponding areas, $S_{1}$ and $S_{2}$, used in Equation (28). The first area, $S_{1}$, is a polygon (filled with vertical stripes in this figure), whereas the second, $S_{2}$, is a rectangle (filled with horizontal stripes here).

**Figure 12:**
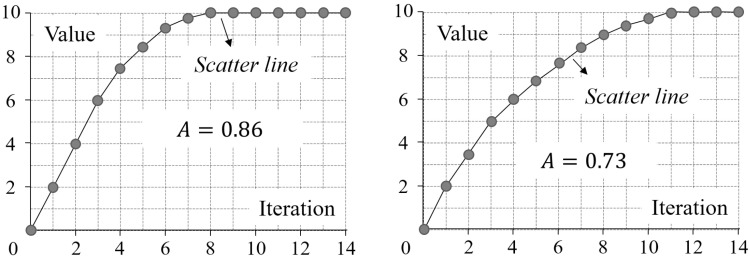
Two example scatter lines and their corresponding values of the ability measure (A).

The ability to capture configurational disorder refers to the range of configurations, in terms of the degree of disorder, that can be captured by an improved Shannon entropy. An improved Shannon entropy of high ability should capture a large range of configurations, say, from (nearly) completely ordered to totally disordered. For the testing images, the values of a high-ability improved Shannon entropy should converge slowly over the iterations of mixing. In contrast, for an improved Shannon entropy of low ability, its values converge quickly. The measure of ability, referred to as A, is defined by the following formula:

(28) $$\{\begin{array}{l} A=S_{1}/S_{2}\\ S_{1}=\overset{n-1}{\underset{i=1}{\sum }}\frac{1}{2}\left[\left(v_{i}-min\right)+\left(v_{i+1}-min\right)\right]\\ S_{2}=\left(n-1\right)\times \left(max-min\right) \end{array}$$

where $v_{i}$, n, max, and min hold the same meaning as in Equation (27). The nature of A is the ratio of areas (i.e., $S_{1}$ and $S_{2}$) of two shapes formed in the scatter plot of the values of an improved Shannon entropy for a sequence of increasingly configuration-disordered images, as shown in Figure 11. A smaller value of this ratio means that the value of an improved entropy converges slower over the iterations of mixing, as shown in Figure 12. Therefore, the smaller this ratio is, the higher ability the improved entropy is.

## 4. Evaluation and Results Analysis

### 4.1. Methods to be Evaluated: Original and Modified

Methods that were evaluated in this study are listed in Table 1. These methods contain the original Shannon entropy and all the improved methods reviewed in Section 2. In addition, some modified improved Shannon entropies are also tabulated in Table 1, namely *Br96-5*, *Qu12-V′*, *Qu12-V-5*, *Qu12-V-5′*, *Qu12-G′*, and *Qu12-L′*. Modifications performed are as follows:

(1) Changing the size of the neighborhood

The two GLV-based improved Shannon entropies, *Br96* and *Qu12-V*, were originally proposed based on the neighborhood of 3×3 pixels. In this evaluation, their values were also computed by using the neighborhood of 5×5 pixels; the results are referred to as *Br96-5* and *Qu12-V-5*, respectively. The size of the neighborhood used in other entropies was not changed because their computation is limited to only the original size; for example, the size of the neighborhood used in computing *Qu12-G* is fixed at 3×3 pixels by the Sobel operator.

(2) Avoiding dividing by zero

There is a problem of dividing by zero in the three improved Shannon entropies by Quweider [37], i.e., *Qu12-V*, *Qu12-G*, and *Qu12-L*, if the busyness $m_{l}$ in Equation (17) takes the value of zero. To fix this problem, the strategy used in *Br96*—adding one to the denominator, as shown in Equation (15)—was adopted in this study. Accordingly, a modified formula to Equation (17) was proposed in this study, as shown in Equation (19). The modified results of *Qu12-V/G/L* and *Qu12-V-5* computed using Equation (29) are referred to as *Qu12-V′/G′/L′* and *Qu12-V-5′*, respectively:

(29) $$H=-\overset{n}{\underset{l=1}{\sum }}p_{l}\cdot \log\left(\frac{p_{l}}{m_{l}+1}\right)$$

### 4.2. Results of the Evaluation

**Figure 13:**
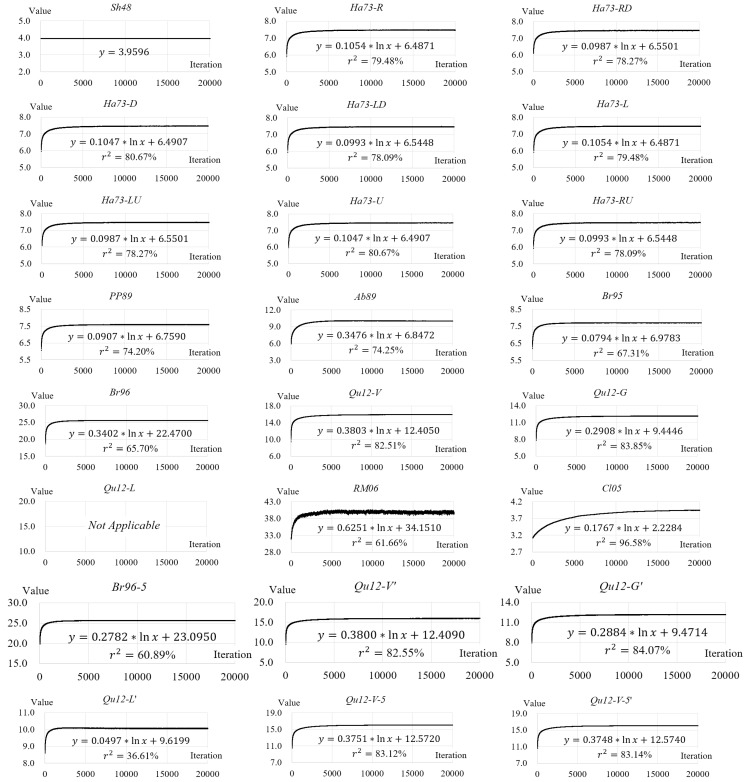
The changes of the 24 entropies along with the 20,000 iterations of mixing of pixels in the natural image.

The entropies of each increasingly configuration-disordered image generated in this study are shown in Figure 13. Note that the logarithmic base in computing each entropy was set as two in this study, although other bases such as 10 and e are also acceptable. Furthermore, this figure shows the results of the regression analysis for each Shannon entropy, namely the regression equation and $r^{2}$. The validity, reliability, and ability, measured by V, R, and A, respectively, of each Shannon entropy are listed in Table 2.

### 4.3. Analysis of the Results on Validity

Among the 23 improved Shannon entropies, only *Qu12-L* and *Qu12-L′* turn out to be invalid in the evaluation, as shown in Table 2. Although both of these improved Shannon entropies are based on LBP, they are invalid due to different reasons.

*Qu12-L* is not valid as its algorithm returned an error of “dividing by zero” when using Equation (17). In other words, the parameter $m_{l}$ in Equation (17) has a chance of taking the value of zero when dealing with the testing images. In fact, this error makes sense when computing *Qu12-L* with any image. According to Equation (21), $m_{l}$ takes the value of zero if the LBP value of each pixel at the gray level of l equals zero, or, in other words, if all the immediate neighbors of the pixels at the gray level of l have a gray value not greater than l. This condition is always true when l equals the greatest gray value when dealing with any image.

**Figure 14:**
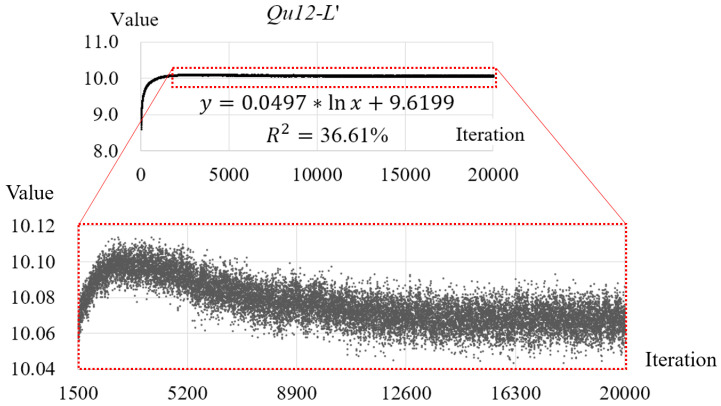
The value change of *Qu12-L′* over the iterations of mixing.

*Qu12-L′* is invalid because its values for the testing images present a convex trend, rather than a logarithmic trend, over the iterations of mixing. This convex trend can be revealed by a close look at the scatter plot of *Qu12-L′*: As shown in Figure 14, the value of *Qu12-L′* first presents an upward trend, peaks at about Iteration 3000, and then shows a downward trend.

### 4.4. Analysis of the Results on Reliability

The ranking of different improved Shannon entropies can be determined according to the measure of reliability (i.e., R), as shown in Table 3. It can be seen from this table that the most reliable improved Shannon entropy is the one based on the average distance between same/different-value pixels, i.e., *Cl05*, followed by the improved Shannon entropies based on GLV, namely *Qu12-V-5*, *Qu12-V-5′*, *Qu12-V*, *Qu12-V′*, *Br96-5*, and *Br96* (ranked 2nd–7th, respectively).

The middle of the rankings is mainly comprised of improved Shannon entropies based on GLCM, containing *Br95* (8th), *Ab89* (9th), *PP89* (12th), and *Ha73-R/L/D/U/LD/RU/RD/LU* (13th–19th). It is noted that there are four pairs of GLCM-based improved Shannon entropies that have the same reliability, namely (a) *Ha73-R* and *Ha73-L*; (b) *Ha73-D* and *Ha73-U*; (c) *Ha73-LD* and *Ha73-RU*; and (d) *Ha73-RD* and *Ha73-LU*. This fact demonstrates that two GLCMs generated based on opposite displacement operators are the same, and it explains why only four, rather than eight, directions are used in *Br95*. It is also noted that the improved Shannon entropies based on the GLCM generated along multiple directions (i.e., *Br95*, *Ab89*, and *PP89*) are more reliable than that based on the GLCM generated along a single direction.

**Figure 15:**
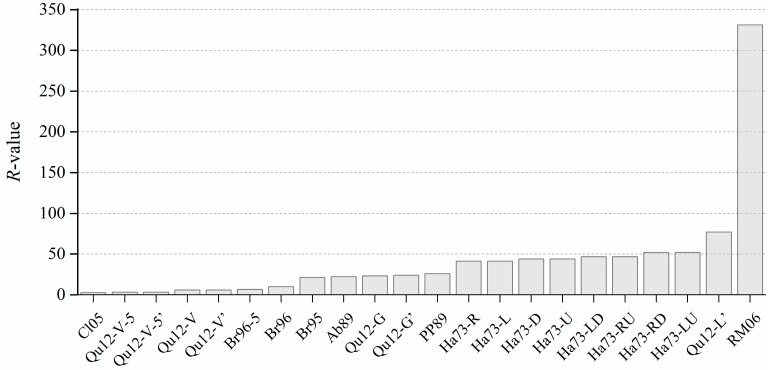
The R-value of various improved Shannon entropies in the ranking order.

The most unreliable improved Shannon entropy is the one based on Laplacian pyramid, i.e., *RM06*, whose R-value is significantly higher than that of the other improved Shannon entropies, as shown in Figure 15. A possible explanation for the low reliability of *RM06* (i.e., the great fluctuation in the value of *RM06*) is that in the mixing simulation, the “motion” of each pixel has a “butterfly effect” on the resultant Laplacian pyramid. In other words, the motion of even a single pixel is enough to change all the levels of the Laplacian pyramid of an image.

### 4.5. Analysis of the Results on Ability

**Figure 16:**
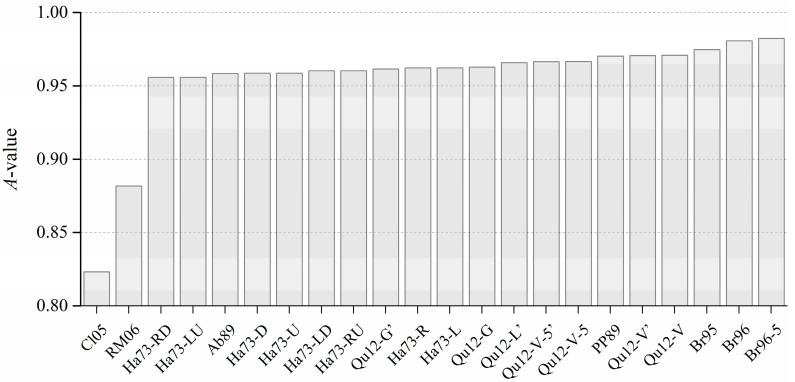
The A-value of various improved Shannon entropies in the ranking order.

The rankings of various improved Shannon entropies in terms of ability is shown in Table 4. It can be seen from the rankings that *Cl05* is the improved Shannon entropy with the highest ability to capture configurational disorder, followed by *RM06* with the second highest ability. In addition, the ability of these two improved Shannon entropies, especially *Cl05*, is significantly better than that of the others, as shown in Figure 16. This significant difference is because these two improved Shannon entropies are sensitive to not only configurations (referred to as local configurations) within a pixel’ neighborhood of a pre-set size but also configurations (global configurations) outside the neighborhood.

**Figure 17:**
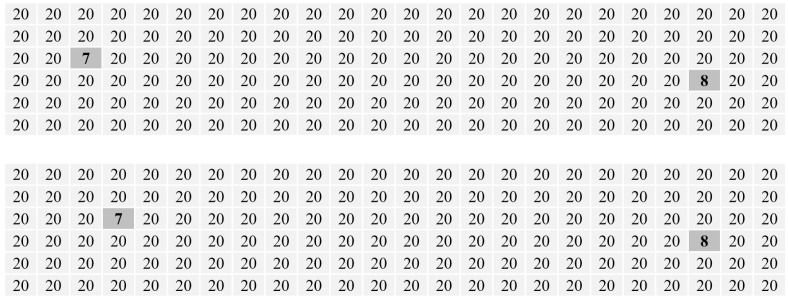
Two simple images with a slight difference.

Let us take the two images (the upper one and the lower) in Figure 17 as an example. The only difference between the two images is the location of the pixel with a gray value of seven. For this pixel, its local configuration within a pre-set size, say 3×3, in the upper image is the same as that in the lower image, but its global configurations are different between the two images (obviously evident in the distance between this pixel and the one with a gray value of eight). The values of all improved Shannon entropies of these two images were computed and are shown in Table 5. One can note from this table that, among all these improved Shannon entropies, only *Cl05* and *RM06* capture the difference between the two images in Figure 17.

## 5. Discussion

### 5.1. Effects of Modifications on Improved Shannon Entropies

In this section, we investigate the effects of modifications on improved Shannon entropies. As described in Section 4.1, the first modification is to change the size of the neighborhood used in computing *Br96* and *Qu12-V*, resulting in two modified improved Shannon entropies, namely *Br96-5* and *Qu12-V-5*. A comparison between the performance of *Br96* and that of *Br96-5* reveals that such a modification increases the reliability but decreases the usability of *Br96*. The changing of the size of the neighborhood, however, improves both the reliability and the usability of *Qu12-V*. These findings imply that neighborhoods of larger sizes are not always better than that of smaller ones in improving Shannon entropy.

The second modification was aimed at avoiding the problem of dividing by zero when computing *Qu12-V*, *Qu12-V-5*, *Qu12-G*, and *Qu12-L*, but this problem was encountered only in the computation of *Qu12-L* in the evaluation (as shown in Figure 13). It is worth noting that although the other three improved Shannon entropies, i.e., *Qu12-V*, *Qu12-V-5*, and *Qu12-G*, are available with the testing images in this study, it does not deny the necessity of this modification. For example, these three improved Shannon entropies are unavailable when dealing with an image where all the pixels have the same gray value.

### 5.2. Computational Efficiency of Various Improved Shannon Entropies

In this section, the computational efficiency of these improved Shannon entropies is discussed. It is necessary to note that an efficiency evaluation (in terms of central processing unit, CPU, time [73]) was not formally included in this study due to two reasons. First, the algorithms of the improved Shannon entropies were implemented in different programming environments in this study. More specifically, the algorithm of *RM06* was implemented in MathWorks (MatLab, R2016a) while that of the other improved Shannon entropies in Visual Studio (Microsoft, 2015). Second, some of the improved Shannon entropy algorithms were optimized in this study to improve their efficiency; otherwise, it takes—according to preliminary estimates—a week with a desktop computer to compute all the improved Shannon entropies of the 20,000 testing images.

To provide an intuitive insight into the computational efficiency of different Shannon entropies, the following experiment was carried out with a desktop computer equipped with an Intel Core i7-4790 CPU @ 3.60 GHz and 8.00 GB RAM. First, a total of 100 configuration-disordered images were randomly selected from the testing image dataset. Then, all the Shannon entropies of each selected image were computed using algorithms without any optimization. The CPU time required by each computation was recorded and is shown in Table 6. It can be seen from this table that *Cl05* is the most time-consuming Shannon entropy.

### 5.3. Nature of the Best-Performed Method: Entropy or Not

**Figure 18:**
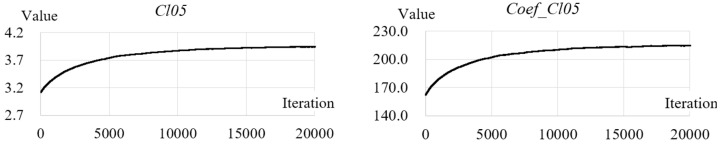
Comparison between the change of *Cl05* and that of *Coef_Cl05* along with the 20,000 iterations of mixing of pixels of the natural image.

**Figure 19:**
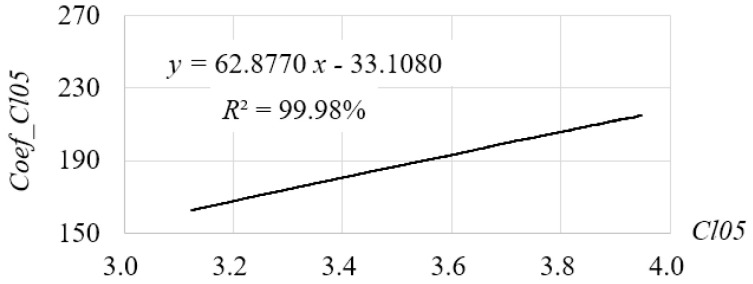
The relationship between *Cl05* and *Coef_Cl05*.

It has been shown in the evaluation that *Cl05* is the best method according to the three criteria defined in this study. However, one may argue that such a method is essentially not a Shannon entropy because it can be replaced by its coefficient, $d_{s}/d_{d}$, which is an index of correlation. Here we first removed the probability component from the equation of *Cl05*, leaving only the coefficients as shown in Equation (30) (referred to as *Coef_Cl05*). Then, we computed the values of *Coef_Cl05* for all the testing images and found that the trend shown by Coef_Cl05 is similar as that of *Cl05*, as shown in Figure 18. A further regression analysis shows that there is a strong liner relationship between *Cl05* and *Coef_Cl05*, as shown in Figure 19:

(30) $$Coef\_Cl05=\overset{n}{\underset{i=1}{\sum }}\frac{d_{s}\left(i\right)}{d_{d}\left(i\right)}$$

### 5.4. Thermodynamic Entropy and Fractal Dimension

It is appropriate at this point to mention two relevant topics, namely thermodynamic entropy and fractal dimension. The concept of thermodynamic entropy, as its name suggests, originates from thermodynamics which is a branch of physics dealing with the movement of energy [74]. Thermodynamic entropy (sometimes referred to as Boltzmann [75] entropy) is similar, or even equivalent in some sense [76], to Shannon entropy, as both of them can be used to statistically characterize the disorder of a system [77,78]. But a clear difference between them is that Shannon entropy is commonly expressed in binary digits per unit (e.g., bits per pixel), while thermodynamic entropy is quantified in units of energy divided by temperature [79].

Although Shannon entropy sometimes is capable of characterizing the disorder of a system, the characterization depends largely on the scale adopted to measure that system (i.e., measurement scale). That is, the value of Shannon entropy may differ largely with the measurement scale. In this sense, one needs to determine the characteristic scale [80,81,82,83] of a system before computing an entropy. However, a large number of systems, such as urban forms and coastlines, are “scale-free” [84,85], namely that they have no characteristic scales. In this case, fractal metrics, such as fractal dimension [86,87], information dimension [88,89], and ht-index [90,91,92,93], can be used as effective alternatives to Shannon entropy because these metrics are independent of measurement scales.

## 6. Conclusions

In this study, a systematic evaluation of various improved Shannon entropies was conducted. In doing so, a critical review was first undertaken on the improvements on Shannon entropy for quantifying the configurational information (i.e., the configurational disorder) of a gray-level image. Next, a systematic evaluation of various improved Shannon entropies was designed. To generate testing data for such an evaluation, a strategy for simulating the mixing of ideal gases—a thermodynamic process of entropy increasing—was proposed in this study. Furthermore, to evaluate the performance of improved Shannon entropies, three criteria were defined (i.e., validity, reliability, and ability to capture configurational disorder) and three measures were developed to assess the fulfillment of each criterion. Finally, 23 variants of Shannon entropy (Table 1) were evaluated, with a testing dataset containing 20,000 increasingly configuration-disordered images. From the results of the evaluation, the following can be concluded:

1. Among all the variants of Shannon entropy, only the two based on LBP (local binary pattern)—*Qu12-L* and *Qu12-L′*—are invalid to quantify the configurational information of an image. However, it is worth noting that, although valid with the testing images in this study, *Qu12-V*, *Qu12-V-5*, and *Qu12-G* may be invalid with other images due to dividing by zero.
2. Variants of Shannon entropy differ significantly in terms of reliability. The most reliable variant of Shannon entropy is *Cl05*, with an R-value of 2.50. In contrast, the least reliable one is *RM06*, with an R-value of 331.23 that is 131 times larger than that of *Cl05*.
3. In terms of the ability to quantify configurational information (i.e., to capture configurational disorder), the best two variants of Shannon entropy are *Cl05* (with an A-value of 0.82) and *RM06* (with an A-value of 0.88). As for the other variants, they have a similar performance with A-values ranging from 0.96 to 0.98.
4. *Cl05* is the best variant of Shannon entropy for quantifying the configurational information of images according to the three criteria defined in this study. However, from a theoretical point of view, it is debatable whether the nature of *Cl05* is still in Shannon entropy or not; from a technical point of view, practical applications of *Cl05* in remote sensing image processing may be limited by its high computational complexity.

The significance of this study can be seen from two perspectives. Theoretically, it presents for the first time a comprehensive evaluation framework (including testing data, criteria, and measures) for the usability of various of entropies. This evaluation framework will play a guiding role in further improving the usability of information-theoretic measures for spatial sciences. Practically, the conclusions of this study are useful for various image processing applications in selecting an entropic measure. For example, a number of band selection algorithms [94,95,96,97] for hyperspectral remote sensing images rely on entropic measures for characterizing the information content of each band. In this case, the improved Shannon entropies which are valid and reliable in this study can be used as effective alternatives to the original Shannon entropy.

Future research is recommended in two areas. First, the computational efficiency of *Cl05* can be improved to achieve its real-time performance with large datasets. For this purpose, some advanced computational means, such as parallel [98,99] and cloud computing [100,101], may be of use. Second, a comparison can be made between the improved Shannon entropies and Boltzmann entropy, which is “both configurational and compositional” [102] and has been recommended for use as an alternative to Shannon entropy in characterizing spatial disorder [31,103].

## Figures and Tables

**Table 1:** A list of Shannon entropies evaluated in this study. These entropies contain the original one by Shannon and twenty-three improved by a number of researchers.

No.	Entropy	No.	Entropy	No.	Entropy	No.	Entropy
1	*Sh48*	7	*Ha73-LU*	13	*Br96*	20	*Br96-5*
2	*Ha73-R*	8	*Ha73-U*	14	*Qu12-V*	22	*Qu12-V′*
3	*Ha73-RD*	9	*Ha73-RU*	15	*Qu12-G*	16	*Qu12-G′*
4	*Ha73-D*	10	*PP89*	19	*Qu12-L*	18	*Qu12-L′*
5	*Ha73-LD*	11	*Ab89*	21	*RM06*	23	*Qu12-V-5*
6	*Ha73-L*	12	*Br95*	17	*Cl05*	24	*Qu12-V-5′*

**Table 2:** The validity (V), reliability (R), and ability (A) of the 24 entropies.

Entropy	V	R	A	Entropy	V	R	A
*Sh48*	No	1	N/A	*Br96*	Yes	9.88	0.98074
*Ha73-R*	Yes	41.21	0.96215	*Qu12-V*	Yes	5.73	0.97068
*Ha73-RD*	Yes	51.70	0.95573	*Qu12-G*	Yes	23.33	0.96266
*Ha73-D*	Yes	43.96	0.95859	*Qu12-L*	No	N/A	N/A
*Ha73-LD*	Yes	46.67	0.96023	*RM06*	Yes	331.23	0.88170
*Ha73-L*	Yes	41.21	0.96215	*Cl05*	Yes	2.50	0.82325
*Ha73-LU*	Yes	51.70	0.95573	*Br96-5*	Yes	6.46	0.98236
*Ha73-U*	Yes	43.96	0.95859	*Qu12-V′*	Yes	5.76	0.97046
*Ha73-RU*	Yes	46.67	0.96023	*Qu12-G′*	Yes	24.13	0.96139
*PP89*	Yes	25.91	0.97016	*Qu12-L′*	No	77.09	0.96572
*Ab89*	Yes	22.43	0.95831	*Qu12-V-5*	Yes	3.03	0.96644
*Br95*	Yes	21.46	0.97460	*Qu12-V-5′*	Yes	3.04	0.96632

Note: N/A means “not applicable”.

**Table 3:** Rankings of various improved Shannon entropies in terms of reliability.

Ranking	Entropy	Ranking	Entropy	Ranking	Entropy	Ranking	Entropy
1	*Cl05*	7	*Br96*	13	*Ha73-R*	19	*Ha73-RD*
2	*Qu12-V-5*	8	*Br95*	**13**	*Ha73-L*	**19**	*Ha73-LU*
3	*Qu12-V-5′*	9	*Ab89*	15	*Ha73-D*	21	*Qu12-L′*
4	*Qu12-V*	10	*Qu12-G*	**15**	*Ha73-U*	22	*RM06*
5	*Qu12-V′*	11	*Qu12-G′*	17	*Ha73-LD*	N/A	*Qu12-L*
6	*Br96-5*	12	*PP89*	**17**	*Ha73-RU*		

Note: Some rankings are bolded to indicate that they are the same as their previous one.

**Table 4:** Rankings of various improved Shannon entropies in terms of ability.

Ranking	Entropy	Ranking	Entropy	Ranking	Entropy	Ranking	Entropy
1	*Cl05*	**6**	*Ha73-U*	13	*Qu12-G*	19	*Qu12-V*
2	*RM06*	8	*Ha73-LD*	14	*Qu12-L′*	20	*Br95*
3	*Ha73-RD*	**8**	*Ha73-RU*	15	*Qu12-V-5′*	21	*Br96*
3	*Ha73-LU*	10	*Qu12-G′*	16	*Qu12-V-5*	22	*Br96-5*
5	*Ab89*	11	*Ha73-R*	17	*PP89*	N/A	*Qu12-L*
6	*Ha73-D*	**11**	*Ha73-L*	18	*Qu12-V′*		

Note: Some rankings are bolded to indicate that they are the same as their previous one.

**Table 5:** The values of all improved Shannon entropies of the two images in Figure 17.

Entropy	Image 1	Image 2	Entropy	Image 1	Image 2
*Cl05*	0.0179	0.0186	*Qu12-G*	N/A	N/A
*RM06*	4.9112	4.4375	*Qu12-L′*	0.2306	0.2306
*Ha73-RD*	0.2874	0.2874	*Qu12-V-5′*	1.7362	1.7362
*Ha73-LU*	0.2874	0.2874	*Qu12-V-5*	1.1636	1.1636
*Ab89*	0.7315	0.7315	*PP89*	0.2614	0.2614
*Ha73-D*	0.2775	0.2775	*Qu12-V′*	1.6104	1.6104
*Ha73-U*	0.2775	0.2775	*Qu12-V*	0.9636	0.9636
*Ha73-LD*	0.2874	0.2874	*Br95*	0.2738	0.2738
*Ha73-RU*	0.2874	0.2874	*Br96*	7.6384	7.6384
*Qu12-G′*	1.8581	1.8581	*Br96-5*	8.1230	8.1230
*Ha73-R*	0.2472	0.2472	*Qu12-L*	N/A	N/A
*Ha73-L*	0.2472	0.2472			

Note: Image 1 and 2 refer to the upper and the lower image in Figure 17, respectively.

**Table 6:** The CPU time required by various Shannon entropies in dealing with 100 randomly selected testing images.

Entropy	Time/s	Entropy	Time/s	Entropy	Time/s	Entropy	Time/s
*Sh48*	0.2	*Ha73-LU*	0.9	*Br96*	2.2	*Br96-5*	3.5
*Ha73-R*	0.9	*Ha73-U*	0.9	*Qu12-V*	2.6	*Qu12-V′*	2.7
*Ha73-RD*	1.0	*Ha73-RU*	0.9	*Qu12-G*	5.2	*Qu12-G′*	4.9
*Ha73-D*	0.9	*PP89*	0.5	*Qu12-L*	3.1	*Qu12-L′*	3.1
*Ha73-LD*	1.0	*Ab89*	0.6	*RM06*	4.7	*Qu12-V-5*	4.0
*Ha73-L*	0.9	*Br95*	0.6	*Cl05*	3651.4	*Qu12-V-5′*	4.0

## References

[1] Kim J., Zeng H., Ghadiyaram D., Lee S., Zhang L., Bovik A.C. (2017). Deep convolutional neural models for picture-quality prediction: Challenges and solutions to data-driven image quality assessment. IEEE Signal Proc. Mag..

[2] Guan J.W., Yi S., Zeng X.Y., Cham W.K., Wang X.G. (2017). Visual importance and distortion guided deep image quality assessment framework. IEEE Trans. Multimed..

[3] Brankov J.G., Yang Y.Y., Wei L.Y., El Naqa I., Wernick M.N. (2009). Learning a channelized observer for image quality assessment. IEEE Trans. Med. Imaging.

[4] Appina B., Khan S., Channappayya S.S. (2016). No-reference stereoscopic image quality assessment using natural scene statistics. Signal Proc. Image Commun..

[5] Wang K.P., Qi G.Q., Zhu Z.Q., Chai Y. (2017). A novel geometric dictionary construction approach for sparse representation based image fusion. Entropy.

[6] Saleem A., Beghdadi A., Boashash B. (2012). Image fusion-based contrast enhancement. EURASIP J. Image Video Proc..

[7] Wang Z., Bovik A.C., Lu L.G. Why is image quality assessment so difficult?. Proceedings of the 2002 IEEE International Conference on Acoustics, Speech, and Signal Processing (ICASSP).

[8] Cadık M. (2008). Perceptually Based Image Quality Assessment and Image Transformations. Ph.D. Thesis.

[9] Liu J., Huang J.Y., Liu S.G., Li H.L., Zhou Q.M., Liu J.C. (2015). Human visual system consistent quality assessment for remote sensing image fusion. ISPRS J. Photogramm. Remote Sens..

[10] Masek J.G., Honzak M., Goward S.N., Liu P., Pak E. (2001). Landsat-7 ETM+ as an observatory for land cover: Initial radiometric and geometric comparisons with Landsat-5 Thematic Mapper. Remote Sens. Environ..

[11] Price J.C. (1984). Comparison of the information content of data from the Landsat-4 Thematic Mapper and the Multispectral Scanner. IEEE Trans. Geosci. Remote Sens..

[12] Niimi T., Maeda H., Ikeda M., Imai K. (2011). Quantification of image quality using information theory. Australas. Phys. Eng. Sci. Med..

[13] Harrie L., Stigmar H. (2010). An evaluation of measures for quantifying map information. ISPRS J. Photogramm. Remote Sens..

[14] Lin Z.J., Deng B. Quantifying degrees of information in remote sensing imagery. Proceedings of the 8th International Symposium on Spatial Accuracy Assessment in Natural Resources and Environmental Sciences.

[15] Lin Z.J., Yao N., Deng B., Wang C.Z., Wang J.H. (2012). Research on differential coding method for satellite remote sensing data compression. Int. Arch. Photogramm. Remote Sens. Spat. Inf. Sci..

[16] Cheng C.X., Lu F., Niu F.Q. (2006). Verification of raster-based map information measurement. Geo-Inf. Sci..

[17] Wu H.Y., Zhu H.J., Liu Y. (2004). A raster-based map information measurement for QoS. Int. Arch. Photogramm. Remote Sens. Spat. Inf. Sci..

[18] Shannon C.E. (1948). A mathematical theory of communication. Bell Syst. Tech. J..

[19] Shannon C.E., Weaver W. (1949). The Mathematical Theory of Communication.

[20] Noorizadeh S., Shakerzadeh E. (2010). Shannon entropy as a new measure of aromaticity, Shannon aromaticity. Phys. Chem. Chem. Phys..

[21] Gregori-Puigjané E., Mestres J. (2006). SHED: Shannon entropy descriptors from topological feature distributions. J. Chem. Inf. Model..

[22] Pielou E. The use of information theory in the study of the diversity of biological populations. Proceedings of the 5th Berkeley Symposium on Mathematical Statistics and Probability.

[23] Thenkabail P.S., Lyon J.G. (2016). Hyperspectral Remote Sensing of Vegetation.

[24] Feixas M., Bardera A., Rigau J., Xu Q., Sbert M. (2014). Information Theory Tools for Image Processing.

[25] Roberts J.W., van Aardt J., Ahmed F. (2008). Assessment of image fusion procedures using entropy, image quality, and multispectral classification. J. Appl. Remote Sens..

[26] Huang J.Y., Zhou Q.M., Wu Z.F. (2016). Delineating urban fringe area by land cover information entropy: An empirical study of guangzhou-foshan metropolitan area, China. ISPRS Int. J. Geo-Inf..

[27] Hu L.J., He Z.Y., Liu J.P., Zheng C.H. (2015). Method for measuring the information content of terrain from digital elevation models. Entropy.

[28] Fan Y., Yu G.M., He Z.Y., Yu H.L., Bai R., Yang L.R., Wu D. (2017). Entropies of the Chinese land use/cover change from 1990 to 2010 at a county level. Entropy.

[29] Goodchild M.F., Duckham M., Goodchild M.F., Worboys M. (2003). The nature and value of geographic information. Foundations of Geographic Information Science.

[30] Tobler W.R. (1997). Introductory comments on information theory and cartography. Cartogr. Perspect..

[31] Cushman S.A. (2016). Calculating the configurational entropy of a landscape mosaic. Landsc. Ecol..

[32] Li Z.L., Huang P.Z. (2002). Quantitative measures for spatial information of maps. Int. J. Geogr. Inf. Sci..

[33] Li Z.L., Liu Q.L., Gao P.C. (2016). Entropy-based cartographic communication models: Evolution from special to general cartographic information theory. Acta Geod. Et Cartogr. Sin..

[34] Sabuncu M.R. (2004). Entropy-Based Image Registration. Ph.D. Thesis.

[35] Haralick R.M., Shanmugam K., Dinstein I. (1973). Textural features for image classification. IEEE Trans. Syst. Man Cybern..

[36] Claramunt C., Cohn A.G., Mark D.M. (2005). A spatial form of diversity. Spatial Information Theory.

[37] Quweider M.K. (2012). Spatial entropy-based cost function for gray and color Image segmentation with dynamic optimal partitioning. Int. J. Comput. Sci. Netw. Secur..

[38] Zhong Y.F., Cao Q., Zhao J., Ma A., Zhao B., Zhang L.P. (2017). Optimal decision fusion for urban land-use/land-cover classification based on adaptive differential evolution using hyperspectral and LiDAR data. Remote Sens..

[39] Ciriza R., Sola I., Albizua L., Álvarez-Mozos J., González-Audícana M. (2017). Automatic detection of uprooted orchards based on orthophoto texture analysis. Remote Sens..

[40] Pal N.R., Pal S.K. (1989). Entropic thresholding. Signal Proc..

[41] Abutaleb A.S. (1989). Automatic thresholding of gray-level pictures using two-dimensional entropy. Comput. Vis. Graph. Image Proc..

[42] Brink A.D. (1995). Minimum spatial entropy threshold selection. IEEE Proc.-Vis. Image Signal Proc..

[43] Mäenpää T. (2003). The Local Binary Pattern Approach to Texture Analysis: Extensions and Applications.

[44] Brink A.D. (1996). Using spatial information as an aid to maximum entropy image threshold selection. Pattern Recognit. Lett..

[45] Dondes P.A., Rosenfeld A. (1982). Pixel classification based on gray level and local “busyness”. IEEE Trans. Pattern Anal. Mach. Intell..

[46] Chen M., Habib A., He H.Q., Zhu Q., Zhang W. (2017). Robust feature matching method for SAR and optical images by using Gaussian-Gamma-Shaped bi-windows-based descriptor and geometric constraint. Remote Sens..

[47] Susaki J. (2012). Segmentation of shadowed buildings in dense urban areas from aerial photographs. Remote Sens..

[48] Sobel I. History and Definition of the Sobel Operator. https://www.researchgate.net/publication/210198558.

[49] Ojala T., Pietikäinen M., Harwood D. (1996). A comparative study of texture measures with classification based on featured distributions. Pattern Recognit..

[50] Li W., Chen C., Su H.J., Du Q. (2015). Local binary patterns and extreme learning machine for hyperspectral imagery classification. IEEE Trans. Geosci. Remote Sens..

[51] Su L.H., Gibeaut J. (2017). Using UAS hyperspatial RGB imagery for identifying beach zones along the South Texas Coast. Remote Sens..

[52] Rakshit S., Mishra A. Estimation of structural information content in images. Proceedings of the Asian Conference on Computer Vision.

[53] Burt P., Adelson E. (1983). The Laplacian pyramid as a compact image code. IEEE Trans. Commun..

[54] Pan J., Wang M., Cao X.H., Chen S.T., Hu F. (2016). A multi-resolution blending considering changed regions for Orthoimage mosaicking. Remote Sens..

[55] Jähne B. (2005). Digital Image Processing.

[56] Sui D.Z. (2004). Tobler’s first law of geography: A big idea for a small world?. Ann. Assoc. Am. Geogr..

[57] Tobler W.R. (1970). A computer movie simulating urban growth in the Detroit region. Econ. Geogr..

[58] Leibovici D.G., Claramunt C., Le Guyader D., Brosset D. (2014). Local and global spatio-temporal entropy indices based on distance-ratios and co-occurrences distributions. Int. J. Geogr. Inf. Sci..

[59] Li X., Claramunt C. (2006). A spatial entropy-based decision tree for classification of geographical information. Trans. GIS.

[60] Wang B.J., Wang X., Huang J.Z., Cao L.B., Srivastava J. (2011). Spatial entropy-based clustering for mining data with spatial correlation. Advances in Knowledge Discovery and Data Mining.

[61] Kim D.-J., Hensley S., Yun S.-H., Neumann M. (2016). Detection of durable and permanent changes in urban areas using multitemporal polarimetric UAVSAR data. IEEE Geosci. Remote Sens. Lett..

[62] Zamburlin P., Lovisolo D., Ariano P., Panero R., Ferraro M. (2009). A quantitative approach to the dynamics of neurite sprouting induced by a neurotrophic factor. J. Neurosci. Methods.

[63] Hill T.L. (1966). Lectures on Matter and Equilibrium.

[64] Gould H., Tobochnik J. (2010). Statistical and Thermal Physics: With Computer Applications.

[65] Agarwal B.K., Eisner M. (2007). Statistical Mechanics.

[66] Vonk M.E., Tripodi T., Epstein I. (2007). Research Techniques for Clinical Social Workers.

[67] Song W.Z., Jia H.F., Huang J.F., Zhang Y.Y. (2014). A satellite-based geographically weighted regression model for regional PM 2.5 estimation over the Pearl River Delta region in China. Remote Sens. Environ..

[68] Finkelstein M.O. (2009). Basic Concepts of Probability and Statistics in the Law.

[69] Bloom M., Fischer J., Orme J.G. (2006). Evaluating Practice: Guidelines for the Accountable Professional.

[70] Rossi P.H., Lipsey M.W., Freeman H.E. (2003). Evaluation: A Systematic Approach.

[71] Joiner A., Reynard S., Mann D. (1995). Scatter Plots: Plain and Simple.

[72] Gao P.C., Liu Z., Tian K., Liu G. (2016). Characterizing traffic conditions from the perspective of spatial-temporal heterogeneity. ISPRS Int. J. Geo-Inf..

[73] Gao P.C., Zhang H., Li Z.L. (2018). An efficient analytical method for computing the Boltzmann entropy of a landscape gradient. Trans. GIS.

[74] Thess A. (2011). The Entropy Principle: Thermodynamics for the Unsatisfied.

[75] Boltzmann L. (1970). Weitere Studien über das Wärmegleichgewicht unter Gasmolekülen. Kinetische Theorie II.

[76] Chen Y.G. (2009). Analogies between urban hierarchies and river networks: Fractals, symmetry, and self-organized criticality. Chaos Sol. Fract..

[77] Chen Y.G. (2010). A new model of urban population density indicating latent fractal structure. Int. J. Urban Sust. Dev..

[78] Bailey K.D., Parra-Luna F. (2009). Entropy systems theory. Systems Science and Cybernetics.

[79] Bekenstein J.D. (2003). Information in the holographic universe. Sci. Am..

[80] Chen Y.G. (2017). Multi-scaling allometric analysis for urban and regional development. Phys. A Stat. Mech. Appl..

[81] Chen Y.G. (2015). Power-law distributions based on exponential distributions: Latent scaling, spurious Zipf’s law, and fractal rabbits. Fractals.

[82] Chen Y.G. (2016). Defining urban and rural regions by multifractal spectrums of urbanization. Fractals.

[83] Chen Y.G. (2012). The rank-size scaling law and entropy-maximizing principle. Phys. A Stat. Mech. Appl..

[84] Chen Y.G., Zhou Y.X. (2008). Scaling laws and indications of self-organized criticality in urban systems. Chaos Sol. Fract..

[85] Liu G., He J., Luo K.T., Gao P.C., Ma L. (2016). Scale-free networks of the earth’s surface. Int. J. Mod. Phys. B.

[86] Mandelbrot B.B. (1967). How long is the coast of Britain? Statistical self-similarity and fractional dimension. Science.

[87] Zhang H., Li Z.L. (2012). Fractality and self-similarity in the structure of road networks. Ann. Assoc. Am. Geogr..

[88] Chen Y.G., Wang J.J., Feng J. (2017). Understanding the fractal dimensions of urban forms through spatial entropy. Entropy.

[89] Chen Y.G., Wang J.J. (2013). Multifractal characterization of urban form and growth: The case of Beijing. Environ. Plan. B Plan. Des..

[90] Gao P.C., Liu Z., Xie M.H., Tian K., Liu G. (2016). CRG index: A more sensitive ht-index for enabling dynamic views of geographic features. Prof. Geogr..

[91] Gao P.C., Liu Z., Liu G., Zhao H.R., Xie X.X. (2017). Unified metrics for characterizing the fractal nature of geographic features. Ann. Am. Assoc. Geogr..

[92] Jiang B., Ma D. (2018). How complex is a fractal? Head/tail breaks and fractional hierarchy. J. Geovis. Spat. Anal..

[93] Jiang B., Yin J.J. (2014). Ht-index for quantifying the fractal or scaling structure of geographic features. Ann. Assoc. Am. Geogr..

[94] Chang C.-I. (2000). An information-theoretic approach to spectral variability, similarity, and discrimination for hyperspectral image analysis. IEEE Trans. Inf. Theory.

[95] Guo B.F., Gunn S.R., Damper R.I., Nelson J.D. (2006). Band selection for hyperspectral image classification using mutual information. IEEE Geosci. Remote Sens. Lett..

[96] MartÍnez-UsÓ A., Pla F., Sotoca J.M., García-Sevilla P. (2007). Clustering-based hyperspectral band selection using information measures. IEEE Trans. Geosci. Remote Sens..

[97] Cao X.H., Han J.G., Yang S.Y., Tao D.C., Jiao L.C. (2016). Band selection and evaluation with spatial information. Int. J. Remote Sens..

[98] Qin C.Z., Zhan L.J., Zhu A.X., Zhou C.H. (2014). A strategy for raster-based geocomputation under different parallel computing platforms. Int. J. Geogr. Inf. Sci..

[99] Gao P.C., Liu Z., Tian K., Xie M.H. (2016). A comparative study of geographical information services in public and private clouds. Asian J. Geoinf..

[100] Gao P.C., Liu Z., Xie M.H., Tian K. (2016). Low-cost cloud computing solution for geo-information processing. J. Cent. South Univ..

[101] Gao P.C., Liu Z., Han F., Tang L., Xie M.H. (2015). Accelerating the computation of multi-scale visual curvature for simplifying a large set of polylines with Hadoop. GIS Remote Sens..

[102] Gao P.C., Zhang H., Li Z.L. (2017). A hierarchy-based solution to calculate the configurational entropy of landscape gradients. Landsc. Ecol..

[103] Cushman S.A. (2015). Thermodynamics in landscape ecology: The importance of integrating measurement and modeling of landscape entropy. Landsc. Ecol..

